# A Clonal Group of Nontypeable *Haemophilus influenzae* with Two IgA Proteases Is Adapted to Infection in Chronic Obstructive Pulmonary Disease

**DOI:** 10.1371/journal.pone.0025923

**Published:** 2011-10-05

**Authors:** Timothy F. Murphy, Alan J. Lesse, Charmaine Kirkham, Huachun Zhong, Sanjay Sethi, Robert S. Munson

**Affiliations:** 1 Division of Infectious Diseases, Department of Medicine, State University of New York at Buffalo, Buffalo, New York, United States of America; 2 Division of Pulmonary and Critical Care Medicine, Department of Medicine, State University of New York at Buffalo, Buffalo, New York, United States of America; 3 Department of Microbiology, State University of New York at Buffalo, Buffalo, New York, United States of America; 4 Department of Pharmacology and Toxicology, State University of New York at Buffalo, Buffalo, New York, United States of America; 5 New York State Center of Excellence in Bioinformatics and Life Sciences, State University of New York at Buffalo, Buffalo, New York, United States of America; 6 Veterans Affairs Western New York Healthcare System, Buffalo, New York, United States of America; 7 Research Institute at Nationwide Children's Hospital, Columbus, Ohio, United States of America; 8 The Ohio State University, Columbus, Ohio, United States of America; Columbia University, United States of America

## Abstract

Strains of nontypeable *Haemophilus influenzae* show enormous genetic heterogeneity and display differential virulence potential in different clinical settings. The *igaB* gene, which encodes a newly identified IgA protease, is more likely to be present in the genome of COPD strains of *H. influenzae* than in otitis media strains. Analysis of *igaB* and surrounding sequences in the present study showed that *H. influenzae* likely acquired *igaB* from *Neisseria meningitidis* and that the acquisition was accompanied by a ∼20 kb genomic inversion that is present only in strains that have *igaB*. As part of a long running prospective study of COPD, molecular typing of *H. influenzae* strains identified a clonally related group of strains, a surprising observation given the genetic heterogeneity that characterizes strains of nontypeable *H. influenzae*. Analysis of strains by 5 independent methods (polyacrylamide gel electrophoresis, multilocus sequence typing, *igaB* gene sequences, P2 gene sequences, pulsed field gel electrophoresis) established the clonal relationship among the strains. Analysis of 134 independent strains collected prospectively from a cohort of adults with COPD demonstrated that ∼10% belonged to the clonal group. We conclude that a clonally related group of strains of nontypeable *H. influenzae* that has two IgA1 protease genes (*iga* and *igaB*) is adapted for colonization and infection in COPD. This observation has important implications in understanding population dynamics of *H. influenzae* in human infection and in understanding virulence mechanisms specifically in the setting of COPD.

## Introduction

*Haemophilus influenzae* is recovered almost exclusively from the human respiratory tract and is thus uniquely adapted to the human host. No animal or environmental reservoirs have been identified. Nontypeable (non encapsulated) strains of *H. influenzae* colonize the upper respiratory tract of most children within the first 3 years of life and cause otitis media in children when bacteria move from the nasopharynx to the middle ear [1], [2], [3], [4], [5]. Nontypeable *H. influenzae* is an unusual cause of infection in otherwise healthy adults. However, in the setting of chronic obstructive pulmonary disease (COPD), the bacterium is a major pathogen that colonizes the lower airways and causes intermittent exacerbations which punctuate the course of the disease [6], [7].

While encapsulated serotype type b strains, which cause invasive disease in children, form a clonal group, nontypeable strains of *H. influenzae* demonstrate enormous genetic heterogeneity among strains [4], [8], [9], [10], [11], [12], [13], [14]. Nontypeable strains of *H. influenzae* have for a long time been viewed as colonizing bacteria whose virulence potential resulted largely from alteration in host defense. However, recent analysis of strains recovered from children with otitis media and from adults with COPD indicate that strains of nontypeable *H. influenzae* display differential virulence potential [15], [16], [17], [18]. The genomes of essentially all strains of *H. influenzae* contain the *iga* gene that encodes a type 1 IgA1 protease. The genomes of strains recovered from adults with COPD have a higher likelihood of having a newly identified gene that encodes a type 2 IgA1 protease (*igaB*) compared to strains isolated from other clinical sources, including middle ear fluid (otitis media) and nasopharynx [15], [19]. This observation suggests that IgA protease B plays a role in the pathogenesis of infection in the setting of COPD.

To elucidate the dynamics of *H. influenzae* colonization of the respiratory tract in COPD, we have been conducting a prospective study of bacterial infection in adults with COPD in which monthly sputum cultures are performed [20], [21]. Characterization of these isolates using a variety of molecular methods confirms the previous observations of a high degree of genetic diversity among strains [11]. However, over the course of the study, we have identified clonally related strains of nontypeable *H. influenzae* that contain the *igaB* gene within this genetically diverse population of strains collected longitudinally over 10 years. This surprising observation has lead to the identification of a subset of strains of nontypeable *H. influenzae* that form a clonally related group that is adapted to colonize and infect adults with COPD.

## Results

### Characterization of strains isolated from sputum of adults with COPD

Adults with COPD were enrolled in a prospective study and made monthly clinic visits during which their clinical status was evaluated and recorded and they produced sputum samples. In addition to the monthly clinic visits, subjects were evaluated and sputum samples were collected when they were suspected of having an exacerbation suggested by a worsening of symptoms. This study has been described in detail previously [20], [21], [22], [23]. From April 1994 through December 2004, a total of 134 independent isolates of *H. influenzae* were identified based on molecular typing using whole bacterial cell lysates subjected to sodium dodecyl sulfate polyacrylamide gel electrophoresis (SDS PAGE) and pulsed field gel electrophoresis (PFGE) of genomic DNA as previously described [20], [21].

These 134 isolates were characterized previously to determine the proportion of strains that contain the *igaB* gene that encodes a recently recognized IgA protease that is present in some strains of *H. influenzae* in addition to the previously identified IgA protease (encoded by *iga*) that is present in essentially all strains. A total of 60 of the 134 strains have the *igaB* gene [19]. This set of 134 strains, representing all independent isolates recovered from a cohort of adults with COPD collected longitudinally over 10 years, was used in the present study. Each isolate is a first acquisition of that strain for each patient.

### Characterization of sequences surrounding *igaB*

**Figure 1:**
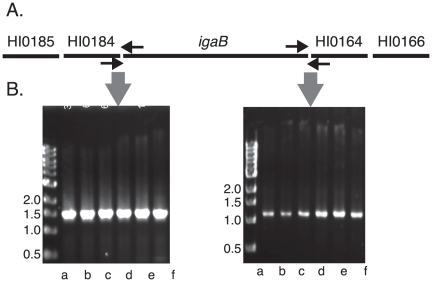
Genes in the region of the *igaB* insertion site. A. Map of genes surrounding *igaB* (not to scale). Gene numbers correspond to numbers in strain Rd KW20 [24]. Horizontal arrows indicate location of oligonucleotide primers used in PCR reactions. B. Ethidium bromide stained agarose gels showing results of PCR reactions with genomic DNA of *H. influenzae* strains that contained the *igaB* gene with the oligonucleotide primers noted in A. Lanes contain genomic DNA from strains a. 3P14H1, b. 6P5H, c. 6P18H1, d. 7P49H1, e. 11P6H, f. 12P37H1. Molecular size standards are noted in kilobases.

Analysis of sequences flanking *igaB* in two strains (11P6H and 2019) revealed that the arrangement of open reading frames (ORFs) in this region of the genome differs from that in strains Rd KW20 and 86-028NP, the first two strains of *H. influenzae* whose genome sequences were determined, and which lack the *igaB* gene [24], [25]. The ORF upstream of *igaB* is HI0184 (nomenclature of strain Rd KW20) and the downstream ORF is HI0164. To determine whether other strains that have the *igaB* gene had similar flanking sequences, PCR reactions using primers noted in Figure 1 were performed. PCR using a primer pair that corresponds to sequences of the 5′ region of *igaB* and the 3′ region HI0184 amplified a fragment from 20 of 20 strains of *H. influenzae* that contain the *igaB* gene (Figure 1). Similarly, PCR using a primer pair that corresponds to the 3′ region of *igaB* and the 5′ region of HI0164 amplified a fragment from 20 of 20 strains that contain the *igaB* gene (Figure 1). Therefore, strains of *H. influenzae* that have the *igaB* gene have the same flanking sequences as one another, indicating that an inversion event occurred at the site of insertion of *igaB*.

**Figure 2:**
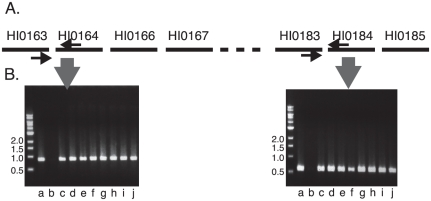
Genes in the region of the *igaB* insertion site. A. Map of genes from ORFs HI0163 through HI0185 (not to scale) in *H. influenzae* strain Rd KW20 which does not contain the *igaB* gene. The dotted line represents ORFs HI0167 through HI0182. Horizontal arrows indicate location of oligonucleotide primers used in PCR reactions. B. Ethidium bromide stained agarose gels showing results of PCR reactions with genomic DNA of *H. influenzae* strains that do not contain the *igaB* gene with the oligonucleotide primers noted in A. Lanes contain genomic DNA from strains a. Rd KW20, b. 11P6H (which contains *igaB*), c. 5P28H1, d. 14P14H1, e. 31P7H1, f. 32P8H1, g. 48P28H1, h. 56P76H1, i. 1862, j. 3222B. Molecular size standards are noted in kilobases.

To determine whether some strains of *H. influenzae* that do not contain *igaB* had a similar genomic inversion, primers were designed to amplify genes predicted to be adjacent to one another if the order of ORFs was the same as that in strain Rd KW20 (i.e., no genomic inversion). PCR reactions using these sets of primers yielded PCR products that would be predicted if the order of ORFs was the same as in strain Rd KW20 in 10 of 10 strains that did not contain *igaB* (Figure 2). Based on this result with 10 clinical isolates and based on the absence of the inversion in the 5 genomes lacking *igaB* that have been sequenced, we conclude that strains of *H. influenzae* in which *igaB* is absent have not undergone the inversion event that is present in strains that contain the *igaB* gene.

**Figure 3:**
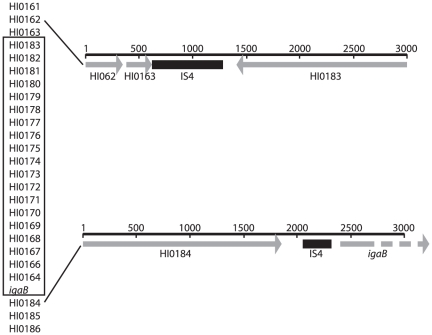
Diagram illustrating the location of the IS4 transposase sequences flanking the genomic inversion in *igaB*-containing strains. The order of the ORFs is noted on the left (strain Rd nomenclature) and the box denotes the inversion. ORFs are noted as gray lines and IS4-like sequences are noted as black lines. Numbers denote base pairs.

Figure 3 shows the arrangement of ORFs in and around the *igaB* gene. The results indicate that a large genomic inversion event occurred at the site of insertion of *igaB* in the *H. influenzae* genome. The origin of the *igaB* gene is considered in more detail below.

### Analysis of *igaB* sequences

The *igaB* gene, previously reported from strain 11P6H, is 5,664 nucleotides in length encoding a protein of 1,887 amino acids [19]. The *igaB* gene from strain 2019 was sequenced as part of the present study. The gene is 4941 nucleotides in length, encoding a protein of 1647 amino acids. The protease domains of both proteins are 86% identical over the first 1028 amino acids. Similarly, the last 285 residues (containing the β-core region of the molecule) are 96% identical. Between these two domains is an alpha helical domain. The IgaB protein from strain 11P6H has 3 repeats in this α helical region; residues 1167 to 1318 are 100% identical to residues 1308 to 1459 and 98% identical to residues 1026 to 1177 [19]. The strain 2019 protein has 2 repeats in this region, accounting for its smaller size. The function(s) of this domain are not well understood but it is thought to function, in part, to stabilize the β-core [26]. The function of the repeats is unknown.

The *igaB* gene was amplified by PCR from 9 additional strains, then sequenced. The derived amino acid sequence of *igaB* from 5 of the 9 strains was identical to that of the derived amino acid sequence of the *igaB* gene of 11P6H (Table 1). The gene from two additional strains contained minor changes in sequence and the final two strains had *igaB* genes containing a frame shift, resulting in a truncated product.

**Table 1:** Multilocus sequence types, IgaB protease sequence and outer membrane protein P2 sequences of *H. influenzae* clonal and related strains.

Strain	MLST type	IgaB amino acid sequence compared to strain 11P6H	P2 amino acid sequence compared to strain 11P6H
11P6H	159	Reference strain	Reference strain
23P2H	159	Not done	Identical
33P46H1	159	Not done	Identical
40P41H1	159	1 amino acid different	Identical
43P2H1	159	Not done	Identical
44P85H1	159	Identical	Identical except 4 amino acid insert
59P3H1	159	Insertion of 4 A's at NT 1272 resulting in truncated gene product	Identical
87P37H1	159	Identical	Identical
102P20H1	159	Identical	Identical
105P9H1	159	Identical	Identical
24P44H1	680	Identical	1 amino acid difference
54P24H1	107	7 amino acids different	96 amino acids different
93P16H1	107	Loss of an A residue at NT 2696 resulting in truncated gene product	96 amino acids different

Among the *igaB* genes we sequenced, the gene encoding the strain 2019 IgaB protein is notable in that downstream of the ATG start codon are 11 repeats of the sequence AAATTCA suggesting that expression of the 2019 IgaB protein is phase variable. The strain 2019 *igaB* gene is flanked on the 5′ side by a homolog of HI0184 and the 3′ side by a homolog of HI0164 as was the case for the gene from strain 11P6H.

A nucleotide BLAST search with *igaB* of strain 11P6H was performed to determine which strains of *H. influenzae* whose genomes are available in GenBank contained *igaB*. Of the six strains of *H. influenzae* whose complete genome has been sequenced, the *igaB* gene is present in one (PittEE) along with the same inversion of ORFs 164 to 183 noted in other strains that have *igaB*. As sequenced, the gene contains a frame shift, so the gene is annotated as a pseudogene (gi|59800725|ref|YP_207437.1| IgA-specific metalloendopeptidase [*Neisseria gonorrhoeae* FA 1090]). Partial genomes of 11 additional strains of *H. influenzae* are also available in GenBank. Given the sequence similarities between *iga* and *igaB*, we used an informatic approach similar in concept to the PCR approach used above to identify the 11P6H-like gene order. Blast searches were performed using the 5′ portion of *igaB* from 11P6H as well as the intergenic region and a portion of the gene upstream of *igaB*. Strains 6P18H1, 7P49H1, 3655 and PittAA had the genomic arrangement observed in 11P6H [19], [27]. Using a similar strategy, we demonstrated that the 11P6H gene arrangement was present 3′ of the *igaB* sequence in these four genomes. Together, these data strongly imply that the *igaB* gene is present in these 4 strains.

### Origin of the *igaB* gene

Blast searches indicate that the *H. influenzae igaB* gene shares more identity with *Neisseria meningitidis iga* genes than the *H. influenzae iga* genes. The region of the *H. influenzae igaB* gene that encodes the secreted portion of the IgA protease (amino acids 1–1020) is 92% identical and 95% similar to *Neisseria* IgA protease while the ß-core domain (amino acids 1529–1887) is 53% identical and 71% similar [15], suggesting that the *Neisseria* and *Haemophilus* genes share a common origin. In further support of this hypothesis, the G+C content of *igaB* is 42.4% which is higher than the G+C content of the *H. influenzae* genome (38 to 38.2%) and *H. influenzae iga* (31.3%) and trending toward that of the *N. meningitidis* genomes, 52% (e!EnsemblBacteria http://bacteria.ensembl.org/Neisseria/index.html). A BLASTX search of the regions flanking *igaB* revealed homology with a portion of an IS4 family transposase 5′ of the *igaB* gene (nucleotides 2065 to 2352 of Genbank Accession DQ423203). If the gene rearrangement was a simple inversion of the region between the homologs of HI0164 and HI0184 with the insertion of *igaB* at one end of the rearranged sequence, the other end of the rearranged sequence would contain a homolog of HI0163 adjacent to a homolog of HI0183. This region from strain 2019 was identified and sequenced. The region between the strain 2019 homologs of HI0163 and HI0183 is 771 nucleotides in length. BLASTX analysis of this sequence demonstrated that 443 nucleotides in this intergenic region had homology to the same IS4-like element (Genbank Accession Yp_002475643, [28]) observed 5′ of *igaB* (Figure 3).

### Identification of a clonal group

The striking homology of the *igaB* genes raised the question of the genetic relationship among strains that contained the gene. To assess the relationship of strains among one another, multilocus sequence typing (MLST) was performed initially on 18 strains isolated from the sputum of adults with COPD as part of our prospective study and whose genomes contained *igaB*. Seven of the strains were MLST type 159, one strain (24P44H1) was MLST 680, which shares 5 of 7 alleles with MLST type 159 and two strains were MLST type 107 which shares a different set of 5 of seven alleles with MLST type 159. The remaining 8 strains had a variety of different MLST types. The identification of 7 strains with the same MLST type was a surprising observation because analysis of thousands of strains of nontypeable *H. influenzae* isolated from our prospective study by SDS PAGE typing and PFGE has revealed a high degree of genetic diversity among nontypeable strains. Each of the 7 MLST type 159 strains was isolated from different patients over a 9-year period. The patients all attended clinics at the Buffalo VA Medical Center but none had direct contact with one another as far as could be determined.

In view of the surprising observation that epidemiologically unrelated strains were the same MLST type, we assessed these relationships using a second independent method. The gene that encodes P2, the major outer membrane porin protein of *H. influenzae*, shows abundant sequence differences among nontypeable strains [29], [30], [31], [32]. Indeed, sequence differences in the P2 gene have been used as a method to distinguish strains of nontypeable *H. influenzae* from one another [21]. Therefore we determined the sequences of the P2 gene of the 7 MLST type 159 strains. The sequence of the P2 genes of the strains that were MLST 159 were identical to one another at the nucleotide level except for strain 44P85H1 which had a 12 base pair insert but was otherwise identical. The P2 gene from strain 24P44H1 had a single nucleotide alteration resulting in a single amino acid alteration compared to the gene from the MLST 159 strains.

In view of this most surprising observation of identical P2 gene sequences among strains that were isolated from different patients at different times over a 10 year period, we assessed the possibility that contaminating DNA in PCR reactions or laboratory error (e.g., strain switches) accounted for the sequence identity. The 7 strains with identical P2 sequences were plated again from the original vials frozen at the first passage after isolation from the sputum sample. The P2 gene was amplified by PCR and the sequence was determined again. These experiments confirmed the previous observation of identical P2 sequences at the nucleotide level.

The P2 gene undergoes point mutations under immune selective pressure during colonization of the respiratory tract in COPD [33], [34], [35]. We determined the P2 gene sequence of 3 pairs of clonal strains upon acquisition and after carriage for 7.4, 16.5 and 19.7 months by COPD subjects. In all 3 instances, the gene sequences remained identical in every nucleotide following colonization of the respiratory of adults with COPD.

The apparent “signature” P2 sequence of the MLST 159 strains was used to screen the remaining isolates, which contained the *igaB* gene, in the set of strains isolated prospectively from adults with COPD over a 10 year period. PCR primers were designed to amplify a fragment from the unique P2 gene of the MLST 159 strains (5′- GCTGTTGTTTATAACAACG and 3′-GTGTTTTTCCTTGCTCAGCTTT) but not from other strains based on analysis of P2 genes in GenBank. Three additional strains (33P46H1, 43P2H1, 87P38H1) yielded PCR products. All 3 were determined to be MLST type 159 and the sequence of P2 genes were identical in every nucleotide to that of strain 11P6H (Table 1). Note that each of the 8 genes that show nearly identical sequences in the clonal strains (P2, *igaB*, the 6 MLST genes) is located in a different region of the *H. influenzae* chromosome.

**Figure 4:**
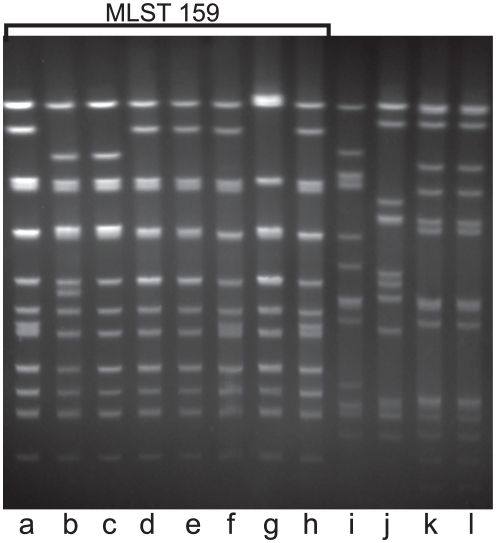
Pulsed field gel electrophoresis of 12 strains of nontypeable *H. influenzae* isolated from the sputum of adults with COPD. Lanes contain genomic DNA cut with *Sma*I from the strains a) 11P6H, b) 44P85H1, c) 87P37H1, d) 102P20H1, e) 105P9H1, f) 23P2H, g) 24P44H1, h) 59P3H1, i) 14P6H1, j) 14P26H1, k) 19P68H7, l) 19P70H8. Strains in lanes a through h belong to MLST 159 as noted.

As a fifth independent method to assess the genetic relationships among strains in this apparent clonal group (in addition to SDS PAGE, *igaB* sequence, MLST and P2 sequence), genomic DNA was subjected to PFGE (Figure 4). Banding patterns were compared using the criteria developed by Tenover et al [36]. The MLST 159 strains were highly related to one another. The minor differences in banding patterns among the MLST 159 strains were consistent with single genetic events such as point mutations. Thus, analysis of the strains by PFGE supports the conclusion that a clonally related group of strains has been identified in adults with COPD.

We conclude that 13 of 134 independent strains of nontypeable *H. influenzae* isolated prospectively from adults with COPD over a 10 year period are clonally related (10 MLST 159 and 3 strains of 2 closely related MLSTs).

### MLST 159 strains

Inspection of the MLST database (http://haemophilus.mlst.net) revealed that 5 strains of MLST 159 have been submitted. All 5 were bloodstream isolates, including 4 recovered as part of the Active Bacterial Core Surveillance program at the Centers for Disease Control (http://www.cdc.gov/abcs/index.html). We acquired those 5 strains and determined the sequence of the gene that encodes P2 of each strain. The sequences were identical to the MLST 159 strains isolated from the adults with COPD in our prospective study.

Various clinical features of the 10 clonal MLST 159 strains were compared to the other 124 strains isolated from the same cohort of COPD patients in the same study (Table 2). The acquisition of a new strain of *H. influenzae* by an adult with COPD may result in an exacerbation or may result in no change in baseline symptoms (colonization) [20], [37]. Acquisition of 4 of the 10 clonal strains (40%) were associated with exacerbation as compared to 59 of 124 (44%) of nonclonal strains (p = 0.64 Chi square). Inclusion of the 3 clonally related strains did not change the result. We conclude that acquisition of a clonal strain was no more or less likely to be associated with exacerbation compared to nonclonal strains.

**Table 2:** Clinical characteristics of patients from whom *H. influenzae* clonal strains were isolated.

Strain^1^	Year Isolated	Clinical Source	Age^2^ (years)	Region of Isolation	Diagnosis	Duration of carriage (mo)
11P6H1	1994	Sputum	64	Buffalo, NY	COPD Exacerbation^3^	1
23P2H	1994	Sputum	64	Buffalo, NY	COPD Colonization^4^	1
33P46H1	2001	Sputum	50	Buffalo, NY	COPD Exacerbation	7
40P41H1	1998	Sputum	66	Buffalo, NY	COPD Colonization	4
43P2H1	1995	Sputum	51	Buffalo, NY	COPD Colonization	3
44P85H1	2003	Sputum	59	Buffalo, NY	COPD Colonization	1
59P3H1	1996	Sputum	45	Buffalo, NY	COPD Colonization	7.4
87P37H1	2002	Sputum	58	Buffalo, NY	COPD Exacerbation	1
102P20H1	2002	Sputum	75	Buffalo, NY	COPD Exacerbation	16.5
105P9H1	2001	Sputum	57	Buffalo, NY	COPD Colonization	19.7
24P44H1^5^	1998	Sputum	53	Buffalo, NY	COPD Exacerbation	1.5
54P24H1^6^	1997	Sputum	81	Buffalo, NY	COPD Exacerbation	1
93P16H1^6^	2000	Sputum	76	Buffalo, NY	COPD Colonization	15.3
M9357	2002	Blood	50	New York	Pneumonia	NA^7^
M10669	2003	Blood	55	Maryland	Pneumonia	NA
M11129	2003	Blood	92	Maryland	Pneumonia	NA
M11131	2003	Blood	45	Maryland	Bacteremia	NA
R3577	1995	Blood	4 mo	Arkansas	Bacteremia	NA

1 Strains are MLST type 159 unless noted otherwise.

2 Age at the time strain was isolated.

3 COPD exacerbation: first isolation of strain simultaneous with clinical exacerbation.

4 COPD colonization: first isolation of strain with no change in baseline symptoms.

5 MLST type 680.

6 MLST type 107.

7 NA: not available.

The duration of carriage, based on molecular typing of prospectively collected strains, was compared for clonal and nonclonal strains of *H. influenzae*. The median duration of carriage of clonal strains did not differ significantly from that of nonclonal strains: 102.5 (40.25–203.5) days (median, 25^th^–75^th^ quartile) for clonal strains compared to 63.0 (33.5–195.25) days for nonclonal strains (p = 0.58 Mann Whitney). Inclusion of the 3 clonally related strains did not change the result. Clonal strains demonstrated variability in the duration of carriage in the cohort of COPD patients (Table 2).

Of the 10 patients who acquired clonal strains, 8 also acquired and carried nonclonal strains of *H. influenzae* at some time during their participation in the prospective study. Thus, individual adults with COPD do not seem to have a specific predilection for clonal strains, indicating that the clonal strains colonize and infect the respiratory tract of adults with COPD as part of the genetically heterogeneous population of nontypeable *H. influenzae*.

## Discussion

In order to survive in the environment of the human respiratory tract, *H. influenzae* relies on the selection and clonal expansion of selected strains rather than on regulatory systems used by many other bacterial species [38], [39]. This strategy accounts in part for the high degree of genetic heterogeneity that is a characteristic feature of nontypeable strains [4], [8], [9], [10], [11], [12], [13]. Several mechanisms facilitate genetic exchange among strains of *H. influenzae* [38]. As a naturally competent bacterium, *H. influenzae* takes up DNA fragments from the environment and inserts the DNA into its genome by homologous recombination. Horizontal exchange also appears to occur by mobile genetic elements as genetically diverse regions of the genome are flanked by phage-related and transposon-like sequences. The present study demonstrates that such mechanisms likely accounted for the acquisition by *H. influenzae* of a second IgA protease gene, *igaB* (essentially all strains contain the *iga* gene) by horizontal transfer from *N. meningitidis*. The homology of *igaB* with meningococcal IgA protease genes and the flanking transposase sequences support this conclusion.

A surprising observation of the present study is the identification of a clonally related group of strains of nontypeable *H. influenzae* that causes infection in the setting of COPD. An extensive literature and the data available in the publicly available MLST database (http://haemophilus.mlst.net) reveal enormous genetic diversity among nontypeable strains of *H. influenzae*. The MLST database currently contains 650 nontypeable strains of ∼1350 total strains that include 814 sequence types. The number of newly recognized sequence types is expanding continuously as more strains are submitted.

The observation that ∼10% of unique strains of nontypeable *H. influenzae* isolated prospectively from the sputum of adults with COPD over a 10 year period belong to a clonally related group suggests that these strains are adapted for colonization of the respiratory tract in this clinical setting. A comparison of various features, including propensity to cause exacerbation and duration of carriage, showed no clear cut differences between clonal and nonclonal strains isolated from our cohort of adults with COPD. While different clinical outcomes following acquisition of clonal strains may be due to differences in selected virulence genes, this result strongly suggests that host factors are important in accounting for differences in the clinical and epidemiological consequences of acquisition of the same clonal strain by different hosts. This conclusion is further supported by the observation that the MLST database contains 5 MLST 159 strains that were isolated from blood and caused invasive infection because many patients who experience invasive infections by nontypeable *H. influenzae* are immunocompromised, [4], [40], [41], [42]. Other determinants that may account for different clinical manifestations of infection with a clonal strain in different hosts include phase variation in the expression of virulence factors and genetic differences, such as genetic islands, that do not affect the housekeeping genes upon which MLST typing is based.

The identification of a clonal group raises the question of whether a strain is being passed from person to person in the cohort. This is an unlikely scenario. All of the strains were isolated from subjects who were living at home in the community. Subjects enrolled in the study have minimal contact with one another. Appointments are scheduled sequentially with one person at a time so subjects are rarely in the waiting room together. The isolates were recovered over a 10 year period with several of the subjects followed in the study separated by years (Table 2). Thus a single strain circulating among patients in the cohort is a highly unlikely explanation for this observation.

Outer membrane protein P2 is a porin protein and is the major surface protein of *H. influenzae* comprising approximately half of the protein content of the outer membrane [43]. P2 has 16 transmembrane β-strands and 8 potentially surface exposed loops which show extensive sequence heterogeneity among strains [29], [30], [31], [32]. P2 is an important target of the human immune response in the setting of COPD [33], [34], [37], [44], [45]. During colonization of the respiratory tract in adults with COPD, the P2 protein undergoes minor amino acid changes in the surface exposed loops of the protein. These amino acid sequence changes result from single base changes in the P2 gene, all generating amino acid changes in the surface-exposed loops, indicating that the changes are driven by immune selective pressure [33], [34], [35]. The observation that the P2 gene sequence of each of the clonal strains is identical in every nucleotide suggests that these strains differ from others in that the P2 gene sequence has remained stable in spite of exposure to the human immune system during colonization and infection. To further explore this observation, the P2 gene sequences of 3 of the clonal strains were determined upon acquisition and after colonization for 7.4, 16.5 and 19.7 months. Each pair of P2 gene sequences remained identical in every nucleotide during these periods of persistence in the human airways.

This high degree of sequence similarity was also observed in the ∼5 kb *igaB* genes in the clonal strains (Table 1) while the *iga* protease gene is quite variable, among strains of *H. influenzae*. We examined the predicted amino acid sequences of the *iga* protease genes from 3 strains isolated from children with otitis media. The IgA protease from strain 86-028NP (accession # YP_248687.1) is 67% identical to the protein from strain R2846 (accession # ADO96711) and 66% identical to the protein from strain PittEE (accession #YP_001291115). The R2846 and PittEE proteases were 70% identical. These numbers are in marked contrast to the near identity of the IgAB protease sequences in clonal strains. *H. influenzae* shows marked variability in its natural competence among strains [46]. We speculate that the clonal strains have evolved mechanisms to maintain the stability of its genome to adapt to the human respiratory tract in COPD, including maintaining the ability to express IgAB protease, given the association of the presence of *igaB* with COPD strains.

*H. influenzae igaB* is homologous with *Neisseria meningitidis iga* that encodes IgA protease and the G+C content of *igaB* is intermediate between that of *H. influenzae* and *Neisseria*. A BLAST search of the regions flanking *igaB* revealed homology with IS4-like transposase both upstream and downstream of the gene [28]. We conclude that *H. influenzae* acquired the *igaB* locus from *N. meningitidis*, an organism that shares the ecological niche of the human upper respiratory tract, based on three observations: 1) the homology between the *igaB* and the meningococcus IgA protease genes, 2) the G+C content of *igaB* that is intermediate between that of *H. influenzae* and *N. meningitidis* and 3) the degenerate transposase sequences flanking *igaB* the 20 kb inversion surrounding *igaB*.

Analysis of sequences that surround *igaB* in strains 11P6H and 2019 revealed the presence of an ∼20 kb inversion (HI0164–HI0183) immediately upstream of *igaB*. Analysis of surrounding sequences in additional strains using PCR showed the presence of the same inversion in 20 of 20 strains that contain the *igaB* gene. Furthermore, PCR analysis of additional strains that lack *igaB* revealed the same gene order in 10 of 10 strains compared to strains Rd and 86-028NP which lack the *igaB* gene. Thus we conclude that the HI0164–HI0183 inversion occurred when *H. influenzae igaB* was acquired from *N. meningitidis* and that this inversion occurred exclusively when the *igaB* was acquired.

Erwin et al [8] analyzed genetic relatedness of *H. influenzae* by MLST and identified 13 clades among 656 strains including 322 nontypeable strains. Sixty percent of nontypeable strains could be placed into one of the 13 clades. There is no obvious phylogenetic clustering of strains in the database based on clinical site of isolation (e.g., middle ear, sputum, blood) or clinical status of the patient. In the course of identifying the clonal group, we determined MLST on 29 independent COPD strains. MLST 159 strains are in clade 13 as are both of the related MLST 107 strains. The remaining 17 isolates showed wide distribution; 4 were each in separate clades. The remaining strains were MLST types that did not fall into any of the defined clades or belonged to new MLST types that were defined subsequent to the analysis published in 2008. Thus the observation that ST 159 clonal strains made up ∼10% of independent strains isolated from adults with COPD indicates that these clonally related strains are over represented in COPD providing support for the concept that these clonally related strains are adapted for colonization and infection of the airways in COPD.

Previous work has established that strains of *H. influenzae* that cause infection in COPD have genome contents that are different from strains that cause infection in other clinical settings. The identification of a clonal group that is adapted for colonization of the airways in COPD provides an opportunity to better understand mechanisms of pathogenesis of infection specifically in COPD. Furthermore, population studies of carefully characterized nontypeable strains should be performed to better understand the population genetics of *H. influenzae*. We speculate that such studies will identify additional clonally adapted groups of strains in the complex population of nontypeable *H. influenzae*.

## Materials and Methods

### Ethics Statement

The study was approved by the Institutional Review Board of the VA Western New York Healthcare System. Written informed consent was provided by study participants. The study was conducted according to the principles expressed in the Declaration of Helsinki.

### Bacterial strains and the COPD Study Clinic

Strains of nontypeable *H. influenzae* were isolated from the sputum of adults with COPD in an ongoing prospective observational study [20]. The study was approved by the Institutional Review Board of the VA Western New York Healthcare System. All subjects provided informed consent. The study was conducted according to the principles expressed in the Declaration of Helsinki. Patients with COPD were seen monthly and at the time of suspected exacerbation. A clinical evaluation using predetermined criteria was performed to assess whether the patient was experiencing an exacerbation or was clinically stable using previously described methods [20]. The determination of whether the patient had stable disease or an exacerbation was made by one of two observers (TFM, SS) and was made before the results of sputum cultures were available.

Sputum samples obtained during these visits were subjected to semiquantitative culture. *H. influenzae* was identified by standard methods and each isolate was subjected to immunoblot assay with monoclonal antibody 7F3 to distinguish from *H. haemolyticus* [11]. When *H. influenzae* was present, 10 colonies were subjected to typing by sodium dodecyl sulfate polyacrylamide gel electrophoresis (SDS-PAGE) as previously described [20]. When analysis by SDS PAGE yielded equivocal results in distinguishing isolates within a patient, strains were subjected to pulsed field gel electrophoresis (PFGE). Isolates from an individual patient were run on the same gel. A strain was considered newly acquired if the unique SDS-PAGE profile was not previously seen in any strains isolated from that patient. Exacerbation strains were defined as newly acquired strains that were initially acquired at the time of clinical evidence of an exacerbation of COPD. Colonization strains were defined as newly acquired strains that were not associated with clinical signs of exacerbation upon initial acquisition of the strain.

Strains M9357, M10669, M11129 and M11131 were provided by the Active Bacterial Core Surveillance of the Emerging Infections Programs Network at the Centers for Disease Control. Dr. Arnold Smith, Seattle Research Institute, provided strain R3577 and Dr. Michael Apicella, University of Iowa, provided strain 2019.

### Multilocus sequence typing

MLST typing was accomplished by determination of the sequences of defined internal fragments of 7 housekeeping genes as previously described [47], [48]. Sequences were submitted to http://haemophilus.mlst.net/ and allele numbers were assigned.

### Pulsed field gel electrophoresis

Selected isolates were subjected to PFGE using previously described methods [21].

### Statistical analysis

Clonal and non-clonal strains of *H. influenzae* were compared for clinical consequences of acquisition, duration of carriage, development of immune response and gap formation. The duration of carriage was non-normally distributed and was compared by the Mann Whitney test. The nominal variables were compared with Chi-square tests.
